# Runs of Homozygosity in Modern Chicken Revealed by Sequence Data

**DOI:** 10.1534/g3.120.401860

**Published:** 2020-10-19

**Authors:** Reza Talebi, Tomasz Szmatoła, Gábor Mészáros, Saber Qanbari

**Affiliations:** * Department of Animal Sciences, Faculty of Agriculture, Bu-Ali Sina University, Hamedan, Iran; † Department of Systems and Synthetic Biology, Agricultural Biotechnology Research Institute of Iran, Agricultural Research, Education and Extension Organization (AREEO), Karaj, Iran; ‡ Centre of Experimental and Innovative Medicine, University of Agriculture in Kraków, Al. Mickiewicza 24/28,30-059 Kraków, Poland; § Department of Animal Molecular Biology, National Research Institute of Animal Production, Krakowska 1, 32-083 Balice, Poland; ** Division of Livestock Sciences (NUWI), University of Natural Resources and Life Sciences, Gregor-Mendel Strasse 33, 1180 Vienna, Austria; †† Leibniz Institute for Farm Animal Biology (FBN), Institute of Genetics and Biometry, 18196 Dummerstorf, Germany

**Keywords:** Inbreeding, ROH islands, White layer, Brown layer, Red Junglefowl

## Abstract

Runs of homozygosity (ROH) are chromosomal stretches that in a diploid genome appear in a homozygous state and display identical alleles at multiple contiguous loci. This study aimed to systematically compare the genomic distribution of the ROH islands among five populations of wild *vs*. commercial chickens of both layer and broiler type. To this end, we analyzed whole genome sequences of 115 birds including white layer (WL, n = 25), brown layer (BL, n = 25), broiler line A (BRA, n = 20), broiler line B (BRB, n = 20) and Red Junglefowl (RJF, n = 25). The ROH segments varied in size markedly among populations, ranging from 0.3 to 21.83 Mb reflecting their past genealogy. White layers contained the largest portion of the genome in homozygous state with an average ROH length of 432.1 Mb (±18.7) per bird, despite carrying it in short segments (0.3-1 Mb). Population-wise inbreeding measures based on Wright’s (F_is_) and genomic (F_ROH_) metrics revealed highly inbred genome of layer lines relative to the broilers and Red Junglefowl. We further revealed the ROH islands, among commercial lines overlapped with QTL related to limb development (*GREM1*, *MEOX2*), body weight (*Meis2a.1*, *uc_338*), eggshell color (*GLCCI1*, *ICA1*, *UMAD1*), antibody response to Newcastle virus (*ROBO2*), and feather pecking. Comparison of ROH landscape in sequencing resolution demonstrated that a sizable portion of genome of commercial lines segregates in homozygote state, reflecting many generations of assortative mating and intensive selection in their recent history. In contrary, wild birds carry shorter ROH segments, likely suggestive of older evolutionary events.

Individuals with a recent common ancestor share sizable part of their genomes identical-by-descent (IBD) state. These individuals transmit IBD segments to their progeny, and create runs of homozygosity (ROH) across the genome (McQuillan *et al.* 2008). ROH were first identified by Broman and Weber in the human genome, whereas Gibson *et al.* acknowledged their importance for inbreeding calculations (Broman and Weber 1999; Gibson *et al.* 2006). McQuillan *et al.* (2008) defined the genomic inbreeding coefficient based on ROH (F_ROH_), which does not depend on allelic frequencies or sampling procedures (Curik *et al.* 2014; Signer-Hasler *et al.* 2017). F_ROH_ was later shown to be a suitable measure for describing levels of inbreeding in breeds with missing pedigree information (Burren *et al.* 2016). Therefore, ROH may provide a more accurate measure of inbreeding levels, compared to the pedigree based measurement (Signer-Hasler *et al.* 2017). While longer haplotypes originate from recent common ancestors, shorter haplotypes inherit from distant ones (background relatedness), and the sum of all these segments are suggested to be an accurate estimation of the inbreeding level of an individual (Ceballos *et al.* 2018a,b; Yoshida *et al.* 2020).

The identification and characterization of ROH islands can further provide insights into population history, structure and demographics over time (Peripolli *et al.* 2017; Signer-Hasler *et al.* 2017). ROH islands are widely observed in populations and can be used as a useful tool to identify the phenomenon called “selective sweeps”, genome regions undergone selection pressure (Szmatoła *et al.* 2019). ROH islands have been used to localize selection signatures in the genome of farm animals including sheep (Purfield *et al.* 2017; Mastrangelo *et al.* 2017; Mastrangelo *et al.* 2018; Luigi-Sierra *et al.* 2019; Signer-Hasler *et al.* 2019), goat (Bertolini *et al.* 2018; Onzima *et al.* 2018), horse (Metzger *et al.* 2015; Grilz-Seger *et al.* 2018; Ablondi *et al.* 2019; Grilz-Seger *et al.* 2019), cattle (Goszczynski *et al.* 2018; Peripolli *et al.* 2018; Nandolo *et al.* 2018; Szmatoła *et al.* 2019; Peripolli *et al.* 2020), pig (Wang *et al.* 2019; Xu *et al.* 2019; Xie *et al.* 2019; Gorssen *et al.* 2020) and in chicken (Marchesi *et al.* 2018; Almeida *et al.* 2019; Strillacci *et al.* 2018; Zhang *et al.* 2018; Zhang *et al.* 2020).

Chicken is a vital livestock for the world food security by producing massive quantities of meat and egg. Chicken also provide an excellent model to investigate the genetics of adaptation, as (1) it involves transformation of the ancestral Red Junglefowl (RJF, *Gallus gallus*), that still runs wild in most of Southeast Asia into a domestic bird. (2) Domestic chicken have experienced intensive selection over the last decades and several breeding companies have independently bred primary multipurpose populations into highly productive birds, so-called broilers (meat-type) and layers (egg-type), by selecting for very similar breeding goals (Elferink *et al.* 2012).

In this study we aimed to systematically compare the genome of domestic birds with each other and against their wild ancestor. The main hypotheses here were to characterize genomic distribution of ROH islands and to localize homozygous segment common among all birds and between broilers *vs*. layers, respectively suggestive of ancient adaptation or footprints of domestication. Our results provide the landscape of homozygosity in sequence resolution, highlighting several candidate genes co-localized with quantitative trait loci (QTL). However, we were unable to pinpoint the standalone ROH islands overlapping among groups of birds as a significant fraction of the genome in modern chicken was carried in homozygous state.

## Material and Methods

### Chicken populations and data preparation

For the purpose of this study we used SNP panel from birds sequenced individually in Qanbari *et al.* (2019). Briefly, it comprises medium coverage (∼10 folds) sequence of the entire genome of 115 chicken samples including white layer (WL, n = 25), brown layer (BL, n = 25), broiler line A (BRA, n = 20), broiler line B (BRB, n = 20) and Red Junglefowl (RJF, n = 25). For further detail of the SNP calling process we refer to the original study. Markers with minimum 1 minor allele were retained for further analysis. To ensure data quality we excluded the SNPs with genotyping rate < 0.1 and significant deviations from the Hardy–Weinberg equilibrium (P_HWE_ <10e-6).

### Measuring diversity metrics

The diversity indicators including observed heterozygosity (Ho), and expected heterozygosity (He), were calculated using PLINK v1.9 package (Purcell *et al.* 2007). Polymorphic marker ratio (PN) that refers to the proportion of polymorphic loci in the target population was also estimated by averaging the proportion of non-missing SNPs per individual for each population.

### Measuring runs of homozygosity

Analysis of ROH was performed using PLINK v1.9 package (Purcell *et al.* 2007). The *–homozyg* module makes ROH calls using a pre-defined sliding window that scans along an individual’s SNP panel to detect homozygous stretches (Howrigan *et al.* 2011). The parameters and thresholds to define an ROH were set according to Almeida *et al.* (2019) and Ceballos *et al.* (2018b). Accordingly, we applied (i) sliding windows of size 50 SNPs across the genome; (ii) the proportion of homozygous overlapping windows set to 0.05; (iii) the minimum number of consecutive SNPs included in a ROH set to 50; (iv) the minimum length of a ROH set to 300 kb; (v) the maximum gap size between consecutive homozygous SNPs set to 1000 kb; (vi) required minimum density to consider a ROH was 1 SNP in 50 Kb; and (vii) a maximum of five SNPs with missing genotypes and up to three heterozygous.

### Measuring inbreeding

Two measures of inbreeding coefficient were calculated with PLINK v1.9 (Purcell *et al.* 2007) for each population.

1. Wright’s inbreeding coefficient (F_is_) was calculated by comparing the difference between observed and expected autosomal homozygous genotypes for each sample as follows:

$$F_{is}=\frac{Number\;of\;observed\;homozygous\;loci-Number\;of\;expected\;homozygous\;loci}{Number\;of\;nonmissing\;loci-Number\;of\;expected\;homozygous\;loci}$$

Genomic inbreeding coefficients based on ROH (F_ROH_) was estimated for each bird according to McQuillan *et al.* (2008). Accordingly, F_ROH_ was defined as:

$$F_{ROH}=\frac{L_{ROH}}{L_{total}}$$

where *L_ROH_* is the total size of ROH in the genome of each bird. *L_total_* is the total size of autosomal chromosomes of chicken covered by SNPs of an individual. The size of autosomal genome was considered as ∼931 Mb according to the chicken reference assembly *Gallus_gallus-5.0.86* available at UCSC Genome Browser. The correlation between the F_ROH_ and F_is_ was calculated for all homozygous stretches and for the five chicken populations. All plots were generated with the R, package ggplot2 for R v3.6.2.

### Distribution of runs of homozygosity

The total number of ROH per chromosome, average length of ROH per chromosome (Mb), and the percentage of chromosomes covered by ROH were estimated in PLINK (*–homozyg* option). Using an in-house R script the identified ROHs were classified into seven length classes of 0.3–1, 1–2, 2–4, 4–8, 8–10, 10–16 and >16 Mb, respectively.

### Detection of autozygosity islands

To investigate genomic regions that were associated with occurrences of ROH within each breed, the fraction of SNPs in ROH was estimated based on the frequency of a SNP in them across individuals. ‘ROH islands’ was identified as a region of adjacent SNPs with an ROH frequency per SNP above the threshold of 1%. These regions were then overlapped with Chicken quantitative trait loci (QTL) database (release 40, 2019, https://www.animalgenome.org/cgi-bin/QTLdb/GG/index).

### Annotation of genetic variants

SnpEff (v.3.4) (Cingolani *et al.* 2012) was used to predict the functional effects of the identified mutations based on reference genome *Gallus_gallus-5.0.86*. The annotated functional categories included: 5 kb up- and down-stream of a gene, intergenic, missense, synonymous, intronic, 3′ untranslated regions, 5′ untranslated regions, stop gain and stop loss. Variants in the up- and down-stream regions and in the 3′ UTR, 5′ UTR regions were merged into the single categories. The bedtools’ (v.2.29.2) (Quinlan and Hall 2010) intersect function was used to determine overlap regions between layers (WL and BL), broilers (BRA and BRB), and commercials (WL, BL, BRA and BRB) within ROH islands.

### Gene-ontology (GO) enrichment analysis

Gene-ontology (GO) enrichment experiment was conducted using the ClueGO plugin v2.1.7 (http://www.ici.upmc.fr/cluego/) (Bindea *et al.* 2009). We further used DAVID v6.8 tool (Huang *et al.* 2009a; Huang *et al.* 2009b) to focus more on significantly enriched Gene GO terms. Only GO/pathway terms with significant enrichment (corrected *P*-value < 0.05 of Benjamini-Hochberg) were included in the analysis.

### Data availability

Data used in this study has been originally published by Qanbari *et al.* (2019) and is deposited in the European Nucleotide Archive (ENA) with the accession number: PRJEB30270. Supplemental material available at figshare: https://doi.org/10.25387/g3.13107176.

## Results and Discussion

### Genotyping, quality control and genetic diversity

The total genotyping rate ranged from 0.97 to 0.99 and the number of polymorphic sites within breeds ranged from 6’100’640 to 14’734’764 (Table 1). RJF was the most diverse population with over 14 million polymorphic sites retained for the final analyses (Table 1). For each breed, genetic diversity was measured using heterozygosity and Wright’s inbreeding coefficient (F_is_). RJF breed, revealed the highest polymorphic marker ratio (99.85%) and despite the lowest observed (H_O_ = 0.240 ± 0.032) and expected heterozygosity (H_E_ = 0.249 ± 0.000) we measured a low level of inbreeding (F_is_ = 0.037) (Table 1). On the contrary, layers represent lowest polymorphic marker ratio and less observed heterozygosity than expected which is attributed to the small number of founders and many generations of mating within closed lines of limited population size, but also partly due to the effect of linked selection (Qanbari *et al.* 2019). Accordingly, F_is_ was markedly high in layers in line with a previous report of genetic diversity and inbreeding in commercial white egg layer line (Bortoluzzi *et al.* 2018). A recent study based on genotypes of a high-density SNP array revealed lower inbreeding (F_is_ = 0.037 to 0.237) in Chinese indigenous chicken breeds than both Europeans local (F_is_ = 0.254 to 0.404) and commercial brown egg layers (F_is_ = 0.144), suggestive of the richer genetic diversity available in indigenous populations (Chen *et al.* 2019).

**Table 1:** Summary statistics of genetic diversity in studied populations

Population	N	NOPS	H_O_ (± SD)	H_E_ (± SD)	%P_N_ (± SD)	Mean F_is_	Max	Min
WL	25	6′100′640	0.278 (±0.020)	0.308 (±0.004)	99.17 (±0.58)	0.102	0.304	−0.028
BL	25	6′199′952	0.282 (±0.033)	0.324 (±0.005)	98.94 (±0.78)	0.135	0.424	− 0.108
BRA	20	10′335′609	0.296 (±0.020)	0.301 (±0.000)	99.83 (±0.03)	0.018	0.212	− 0.041
BRB	20	9′456′158	0.309 (±0.009)	0.309 (±0.000)	99.83 (±0.04)	− 0.0005	0.050	0.047
RJF	25	14′734′764	0.240 (±0.032)	0.249 (±0.000)	99.85 (±0.05)	0.037	0.314	− 0.174

Number of animals (N), number of polymorphic sites (NOPS), average observed heterozygosity (H_O_), average expected heterozygosity (H_E_), polymorphic marker ratio (P_N_) and mean Wright’s inbreeding coefficient (F_is_) values (minimum and maximum) for the individual samples of WL (white Layer), BL (brown Layer), BRA (broiler line A), BRB (broiler line B), and RJF (Red Junglefowl).

### Runs of homozygosity

**Figure 1:**
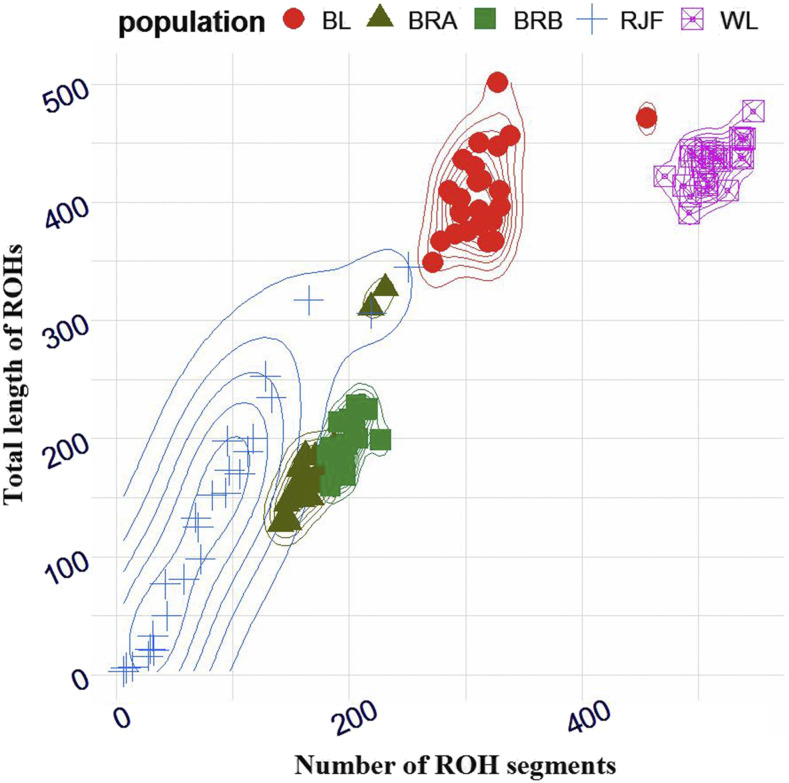
A schematic representation of ROH profile in chicken genome. The profile is given by the total number of homozygous segments and total segment size (Mb). Populations are coded as RJF = red jungle fowl, BL = Brown layer, WL = White layer, BRA = Broiler line A and BRB = Broiler line B.

As schematically shown in Figure 1, distinctive clustering patterns reflected the genetic diversity of five chicken populations. The ROH profile (clusters, Figure 1) in wild birds displayed a more variable pattern than domestic birds (Figure 1). Commercial lines exhibited similar average cumulative size as well as average ROH number, indicating extremely homogenous genomes with low genetic diversity (Table 1).

Descriptive summary of ROH number and length classes is presented in Table 2. As expected, White layers carry more ROH with an average number of 512.28 ± 18.76 per bird (ranging from 547 to 471). By contrast, RJFs encompass least ROH average in 84.00 ± 61.02 (ranging from 251 to 6) per bird (see Table 2 and File S1). The average size of ROH tracts observed in Red Junglefowls is comparable to the African indigenous chicken populations from Rwanda and Uganda both genotyped with the chicken Affymetrix 600K Axiom Array (Elbeltagy *et al.* 2019). Our measure of ROH in broiler populations confirms the dimension reported in previous studies (Marchesi *et al.* 2018; Almeida *et al.* 2019). Layers, especially WL carry the lengthy ROH tracts with 432.1 Mb (± 18.7 Mb; max. 477.1 Mb; min. 390.8 Mb) per bird, whereas the corresponding value was 134.6 Mb (±102.2 Mb; max. 345.4 Mb; min. 3.3 Mb) in wild birds (see File S1). However, the collective length of ROH in WL involved plenty of shorter segments (ROH _0.3-1Mb_, 73.9%) compared to other breeds (Table 2). The largest proportion of the long tracts (ROH _>10Mb_) was found in BL, reflecting recent inbreeding (Table 2). RJF also revealed several long segments of ROH (Table 2) consistent with the observations in indigenous breeds from Netherlands (Bortoluzzi *et al.* 2018), Mexico (Strillacci *et al.* 2018), and China (Zhang *et al.* 2018). These long ROH tracts likely represent the severe bottlenecks, as traditional populations have experienced a drastic reduction in the effective population size in the recent history and genetic experiments show a slow recovery, despite the potential increase in recent population size (Charlesworth 2009).

**Table 2:** Summary statistics of the runs of homozygosity (ROH) based on length classes

	Chicken population				
Class of ROH	Layers		Broilers		Wild birds
	WL (n= 25)	BL (n= 25)	BRA (n= 20)	BRB (n= 20)	RJF (n= 25)
0.3-1Mb	9458 (73.9%)	4631 (58.8%)	2352 (70%)	2820 (71.1%)	1083 (51.6%)
1-2Mb	2619 (20.4%)	1866 (23.7%)	593 (17.6%)	718 (18.1%)	504 (24.0%)
2-4Mb	688 (5.4%)	1001 (12.7%)	327 (9.7%)	350 (8.8%)	330 (15.7%)
4-8Mb	42 (0.3%)	335 (4.3%)	79 (2.3%)	77 (1.9%)	153 (7.3%)
8-10Mb	0	23 (0.3%)	6 (0.2%)	4 (0.1%)	17 (0.8%)
10-16Mb	0	15 (0.2%)	5 (0.1%)	0	12 (0.6%)
>16Mb	0	1 (0.01%)	0	0	1 (0.05%)
Total N. *^a^*	12807	7872	3362	3969	2100
Mean N.*^b^*	512.28	314.88	168.1	198.45	84
Total L. (Mb) *^c^*	10802	10189	3526	3923	3365
Max. (Mb) *^d^*	7.04	21.83	13.85	9.85	17.92
Min. (Mb) *^d^*	0.30	0.30	0.30	0.30	0.30

a Total N.: Total number of ROH detected in the population.

b Mean N.: average number of ROH per individual calculated as the Total N. divided by the number of individuals.

c Total L.: Total length of ROH detected in the population.

d Max, Min.: respectively revealed maximum and minimum length of ROH segments among the total number of ROH.

**Figure 2:**
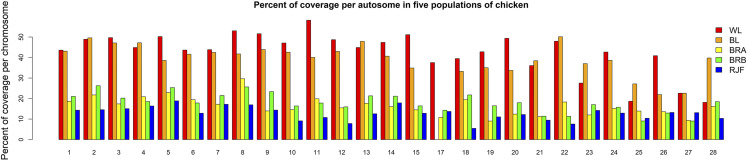
Average percentage of chromosome coverage by runs of homozygosity of minimum length of 0.3 Mb. Populations are coded as WL = White layer, BL = Brown layer, BRA = Broiler line A, BRB = Broiler line B, RJF = Red Junglefowl.

The effect of chicken genome heterogeneity in forming ROH segments was also investigated through comparison of ROH on macro- (GGA1 to 5), intermediate- (GGA6 to 10) and microchromosomes (GGA11 to 28) (Figure 2 and Supplementary Table S1). The GGA16 chromosome did not present any ROH segment due to the assembly fallacy. We found the longest ROH segments on macrochromosomes in line with the lower rate of nucleotide diversity and recombination rate (Axelsson *et al.* 2005; Groenen *et al.* 2009). Overall, macrochromosomes showed the highest number of common ROH and the longest ROH tracts per individual (Supplementary Table S1). The highest percentage of genome covered by ROH in layers was observed in micorchromosomes *e.g.*, in GGA11 (58.21 in WL) and GGA22 (50.12 in BL), (see Figure 2 and Supplementary Table S1), whereas the corresponding chromosomes in broilers and Red Junglefowl were *e.g.*, GGA2 (26.25 in BRB) and GGA5 (18.89 in RJF) (see Figure 2 and Supplementary Table S1).

### Genomic inbreeding coefficient estimated From ROH (F_ROH_)

In the absence of pedigree information, numerous studies have documented the usefulness of ROH segments to infer the inbreeding level of an individual (*e.g.*, Purfield *et al.* 2017; Marchesi *et al.* 2018; Strillacci *et al.* 2018; Zhang *et al.* 2018, among others). We calculated the genomic inbreeding coefficient (F_ROH_) corresponding to the different length classes of ROH segments (Table 3). Consistent with Wright’s inbreeding coefficient, WL was the most inbred population with an F_ROH_ = 0.46 ± 0.02 (see Table 3). The genomic inbreeding coefficient ranked in the following order WL > BL > BRB > BRA > RJF for the populations (see Table 3 and File S1), which is consistent with the Wright’s definition of inbreeding as well as the diversity measures in these populations (*e.g.*, Qanbari *et al.* 2019). Layer lines exhibited markedly greater level of inbreeding in comparison to the broilers and wild birds (Table 3). However, extent of ROH tracts observed in WL and BL is to some extent contradictory. While, a higher proportion of short ROH (F_ROH 0.3−4Mb_) identified in WL (Table 2 and 3), BL carry a higher proportion of long tracts (F_ROH >16Mb_), that might misinterpreted as an ancient origin of inbreeding in layers than broilers. In fact, given the recent genealogy of close mating, layers are expected to carry long homozygosity tracts, as is reported in the previous studies based on array genotypes (Bortoluzzi *et al.* 2018). However, long ROHs tend to break into shorter tracts due to the presence of frequent heterozygous gaps in sequencing resolution (*e.g.*, Qanbari 2020; Szmatola *et al.* 2020), whereas microarrays would likely miss them.

**Table 3:** Average genomic inbreeding coefficient (F_ROH_) for different length categories of ROH across five chicken populations

	Chicken population				
Length category (Mb)	Layers		Broilers		Wild birds
	WL (n= 25)	BL (n= 25)	BRA (n= 20)	BRB (n= 20)	RJF (n= 25)
F_ROH_(0.3-1)	0.228	0.113	0.068	0.082	0.026
F_ROH_(1-2)	0.153	0.113	0.044	0.054	0.031
F_ROH_(2-4)	0.075	0.119	0.048	0.051	0.039
F_ROH_(4-8)	0.009	0.075	0.022	0.022	0.034
F_ROH_(8-10)	0.000	0.009	0.003	0.002	0.007
F_ROH_(10-16)	0.000	0.008	0.003	0.000	0.007
F_ROH_(>16)	0.000	0.001	0.000	0.000	0.001
Total F_ROH_(≥0.3)	0.46	0.44	0.19	0.21	0.14
SD	0.02	0.04	0.05	0.02	0.10

F_ROH_ ≥0.3Mb = overall genomic inbreeding (average of individuals) at ROH threshold of 0.3 Mb, SD = standard deviation of the total F_ROH_ calculated based upon individual F_ROH_, which has been shown in File S1.

**Figure 3:**
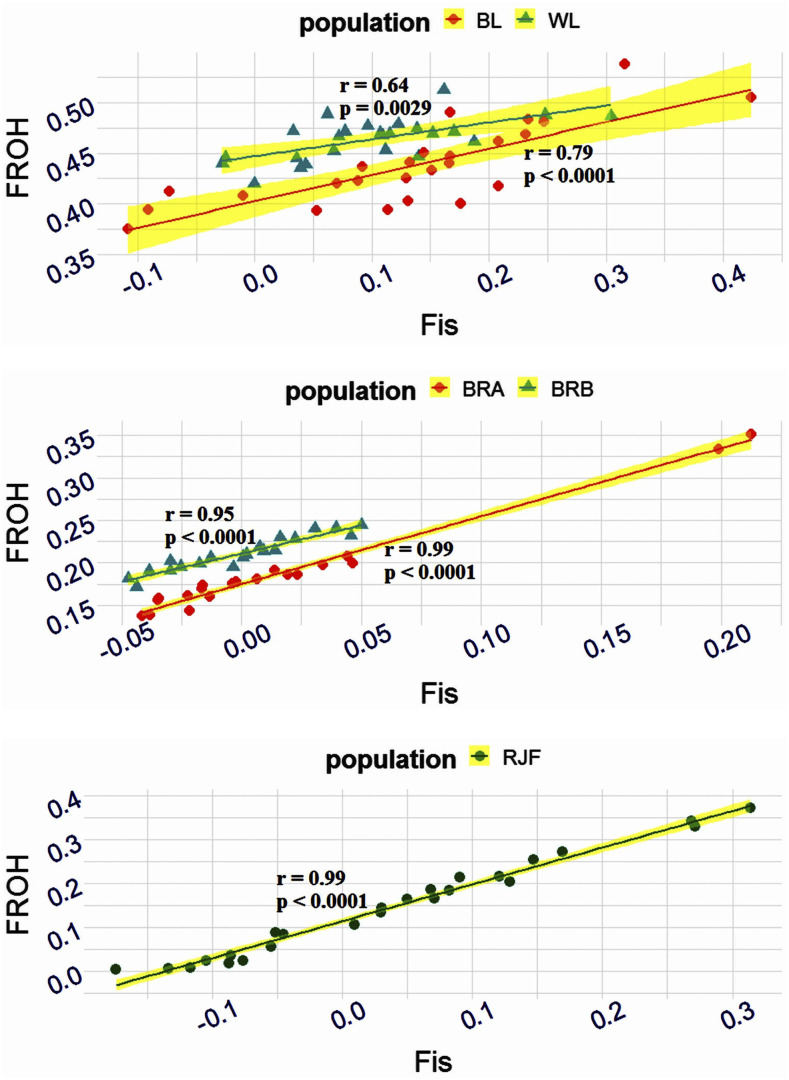
Inbreeding within chicken populations. The Pearson correlation is presented between two measures of molecular inbreeding metrics. Populations are coded as WL = White layer, BL = Brown layer, BRA = Broiler line A, BRB = Broiler line B, RJF = Red Junglefowl.

Figure 3 presents a schematic visualization of Pearson correlation between two measures of molecular inbreeding (F_is_ and F_ROH_). A moderate to strong correlation was found between F_is_ and F_ROH_ within all populations, demonstrating that the extent of a genome under ROH can be used fairly accurately to predict the IBD fraction. The correlation between F_is_ and F_ROH_ was lowest in WL (r = 0.64, *P*-value = 0.0029) and BL (r = 0.79, *P*-value < 0.0001), while the highest correlation was observed in RJFs (r = 0.99, *P*-value < 0.0001). This trend indicates a positive association of the short ROHs with F_is_ and likely suggestive of ancestral inbreeding in wild birds. Studies have reported high correlation between F_is_ and F_ROH_ based on short ROH segments in cattle, suggesting ancestral inbreeding back up to 50 generations ago (Ferenčaković *et al.* 2013; Mastrangelo *et al.* 2016; Limper 2018). The high correlation observed between two metrics of molecular inbreeding is consistent with the previous reports on chicken (Bortoluzzi *et al.* 2018; Marchesi *et al.* 2018; Almeida *et al.* 2019), cattle (Ferenčaković *et al.* 2013; Zhang *et al.* 2015; Mastrangelo *et al.* 2016; Peripolli *et al.* 2018) and sheep (Mastrangelo *et al.* 2017; Purfield *et al.* 2017; Mastrangelo *et al.* 2018) as well as human model (Clark *et al.* 2019). These results confirm the usefulness of the ROH analysis in monitoring differentiation and inbreeding values for further exploitation in chicken breeding programs in the absence of pedigree records.

### ROH islands indicative of selection sweeps

**Figure 4:**
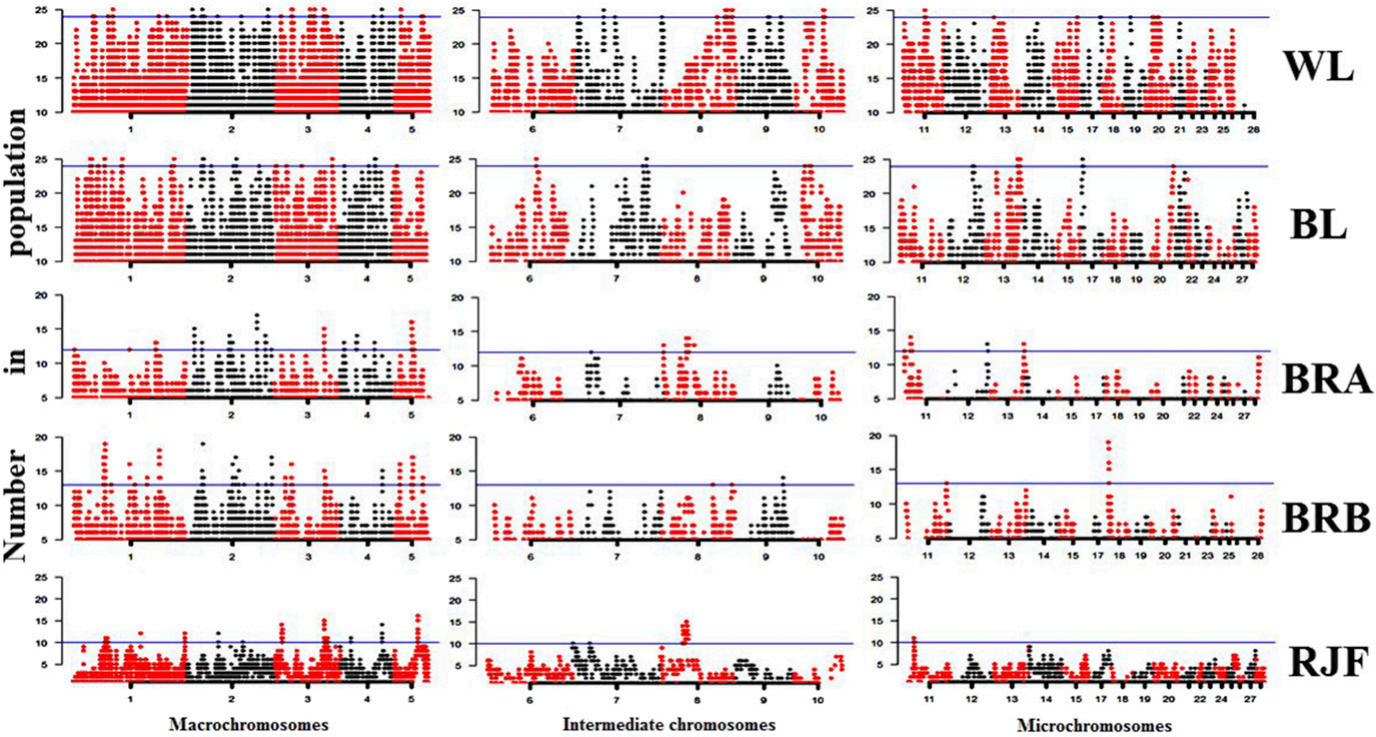
Genome wide distribution of runs of homozygosity (ROH) hotspots. The x-axis represents the SNP genomic coordinate chromosome-wise, and the y-axis shows the proportion of overlapping ROH shared among individuals based upon number in population. Populations are coded as WL = White layer, BL = Brown layer, BRA = Broiler line A, BRB = Broiler line B, RJF = Red Junglefowl.

ROH islands might be indicative of genomic regions underwent natural and/ or artificial selection. We sought to identify most homozygote variants within ROH islands as candidates of recent adaptation and focused on outlaying SNPs in top 1% of ROHs in each population. Given the variable polymorphism content, homozygosity threshold to call an ROH island were population-specific. For example, 96% of the white layers were homozygote in top 1% of the ROH islands, whereas the corresponding figure in wild birds was only 40%. Accordingly, we set the SNP homozygosity thresholds as > 96%, 96%, 60%, 65%, and 40% in WL, BL, BRA, BRB, and RJF, respectively (Figure 4).

We identified 26 to 58 regions of the genome with a high frequency of ROH occurrence, also known as ROH islands, within individuals of particular breeds. ROH islands were detected more frequent in WL (n = 58) and the less in RJF (n = 26). The ROH islands had the average length in range of 223.5 kb ± 177.2 (WL) to 503.8 kb ± 416.6 (BRA), and average number of consecutive SNPs in range 478.3 ± 742.0 (WL) to 4947.3 ± 7220.3 (RJF). The average length of the ROH islands calculated for all breeds was 405.9 kb ± 103.2, while the average number of consecutive SNPs per region was 2108.6 ± 1576.4. Therefore, ROH islands tended to break into shorter segments (see WL in File S2), due to the presence of short heterozygous gaps within the ROH sequences (Nandolo *et al.* 2018). A summary statistics is presented in details in File S2 and shown schematically in Figure 4.

We found 18 ROH islands uniquely detected in wild birds (see Table 4 and File S2). Among the ROH islands, one and seven overlapping regions were detected respectively in layers and broilers out of which three regions in broilers reported to be in association with traits of economic interest available at the Chicken QTL database (release 40). These regions include genes related to antibody response to Newcastle virus (*ROBO2*), eggshell color (*GLCCI1*, *ICA1*, *UMAD1*), antibody titer to SRBC antigen, body weight at 56 days (*Meis2a.1*, *uc_338*) and feather pecking (Table 4). In the same way, the ROH islands detected in RJFs overlapped 53 QTL, among them are genes associated with eggshell strength and eggshell thickness (Table 4). The localized panels of ROHs were further compared with the putative selection signatures reported in Qanbari *et al.* (2019). Our comparison revealed overlap in only two ROH islands located on GGA2 and 5 (see Table 4). The genes located within ROH islands and detected in multiple populations have been presented in File S3.

**Table 4:** Overlapping region among the ROH islands across commercials (layers, broilers) and Red Junglefowl

Chicken group	CHR	Start position (bp)	Stop position (bp)	Number of SNPs	Size (bp)	Associated QTL	Associated Gene	Overlapping region with Qanbari *et al.* 2019*^a^*
Layers	2	27826712	29293102	63	1466.4			
Broilers	1	96797489	97225788	1806	428.5	QTL:24387 “Antibody response to Newcastle virus”	ROBO2	
	2	24570640	25113952	2198	217.3	QTL:24925 “Eggshell color”	GLCCI1, ICA1, UMAD1	
	2	25685016	26072852	1515	387.8	QTL:24961 “Eggshell color”	GLCCI1, ICA1, UMAD1	
	2	133839172	134258895	994	419.7			
	2	143422482	143691103	1602	268.6			RJFs/Coms, RJFs/LRs
	3	84024798	84360723	1401	335.9			
	5	30060267	31863497	7508	1803.2	QTL:153752 “Body weight 56 days”; QTL:14402 “Antibody titer to SRBC antigen”; QTL:15656 “Feather pecking”	Meis2a.1, uc_338	RJFs/Coms, RJFs/BRs, BRs/LRs
Red Junglefowl	1	56231495	56890000	11264	658.5			
	1	57324733	59237097	29851	1912.4	QTL:57888 “Eggshell strength”; QTL:24832 “Visceral peritoneum pigmentation”	ENSGALG00000034601, DNM1L, ENSGALG00000032288, ENSGALG00000032522, PTN, ENSGALG00000029704, CREB3L2, ENSGALG00000030026, DENND5B, ENSGALG00000037580, ENSGALG00000033096, IPO8, ENSGALG00000040988, CCDC77, WEE2, ENSGALG00000037563, C7orf73, SLC13A4, IQSEC3, ENSGALG00000039636, SSBP1, TMTC1, ENSGALG00000027056, FGD4, ENSGALG00000012947, gga-mir-1593, SLC6A12 KDM5A, gga-mir-490, ENSGALG00000019291, WASH1, ENSGALG00000035394, KIAA1147, FAM180A BICD1, gga-mir-6700, PKP2, ENSGALG00000012958, DGKI, AGK, ENSGALG00000035141, SYT10, AMN1 SLC6A13, MTPN, B4GALNT3, CHRM2, ENSGALG00000012969, NINJ2, ENSGALG00000034403, ENSGALG00000034718, ENSGALG00000012919, FAM60A	
	1	59262386	60460076	24311	1197.7	QTL:57881,”Eggshell strength”; QTL:57882,”Eggshell strength”; QTL:57886,”Eggshell strength”; QTL:57887,”Eggshell strength”; QTL:57888,”Eggshell strength”; QTL:57892 “Eggshell thickness”; QTL:57893,”Eggshell thickness”; QTL:57894 “Eggshell thickness”; QTL:57895 “Eggshell thickness”; QTL:57896 “Eggshell thickness”		
	1	116333453	116675094	3428	341.6			
	1	191191672	191740607	5886	548.9	QTL:64552 “Feed intake”		
	2	52312949	52315876	55	2.9			
	2	52841836	52869770	24	27.9			
	2	94716992	94969646	2337	252.7			
	2	95070084	95126392	676	56.3			
	3	11705822	11714324	23	8.5			
	3	12219370	12232316	120	12.9			
	3	86109072	86406883	3322	297.8			
	3	89916285	90709757	7549	793.5			
	4	18397372	18778149	923	380.8	QTL:24914 “Average daily gain”		
	5	41644677	42139793	3630	495.1			
	5	42277347	42279153	7	1.8			
	5	42432216	42799441	4007	367.2			
	7	274155	653362	3321	379.2	QTL:24982 “Age at sexual maturity”		

a Overlapping ROH islands with the putative selective sweeps identified in Qanbari *et al.* 2019. RJFs/Coms: Red Junglefowls *vs*. Commercials; RJFs/LRs: Red Junglefowls *vs*. Layers; RJFs/BRs: Red Junglefowls *vs*. Broilers; BRs/LRs: Broilers *vs*. Layers.

We further conducted an enrichment test on the gene list located in ROH islands to identify over-represented Gene Ontology (GO) terms (Table 5). In commercial lines, genes located in ROH islands were associated with biological functions related to chicken domestication and evolution such as limb development (*GREM1*, *MEOX2*) and negative regulation of apoptotic process (*AVEN*, *ASNS*, *GREM1*) (Table 5). In contrast, the most significant GO term in Red Junglefowl was related to the reproductive traits such as oogenesis (*PTN*, *WASH1*, *WEE2*). These findings show candidate pathways associated with economically important traits and chicken genetic diversity during domestication and recent improvement.

**Table 5:** Functional enrichment of gene ontology terms among the identified genes within ROH islands

Breed	Biological Processes	GO term	Gene
Commercials	limb development	GO:0060173	GREM1, MEOX2
	negative regulation of apoptotic process	GO:0043066	AVEN, ASNS, GREM1
	cell-cell signaling	GO:0007267	GREM1, TAC1
Layers	cell differentiation	GO:0030154	AGR2, AGR3, BZW2
	negative regulation of cell death	GO:0060548	AGR2, AGR3
Broilers	ureteric bud development	GO:0001657	FMN1, GREM1, ROBO2
	cell migration involved in sprouting angiogenesis	GO:0002042	GREM1, SPRED1
Red Junglefowl	oogenesis	GO:0048477	PTN, WASH1, WEE2
	amino acid transmembrane transport	GO:0003333	CGTL, SLC6A12, SLC6A13
	organic acid:sodium symporter activity	GO:0005343	SLC13A4, SLC6A12, SLC6A13

## Conclusion

This study provided the first systematic comparison of runs homozygosity islands between wild and domestic birds in sequence resolution. We found larger fraction of layers genome segregating in homozygote state, reflecting the recent inbreeding in commercial lines, although some of the long ROH tend to break into smaller tracts. Compared to the homogenous values reported for the commercial lines, wild birds showed important variation in the total length of ROH. Regions with a high frequency of ROH occurrence within domestic birds were co-localized with genes implicated in biological functions related to chicken domestication such as a limb development (*GREM1*, *MEOX2*), whereas in Red Junglefowl these regions overlapped with genes related to oogenesis. We also found a modest to high correlation between two molecular measurements, substantiating a highly inbred nature of domestic birds relative to their wild ancestors.
